# Phenotypic Characterisation of Uropathogenic Escherichia coli: Virulence Factors, ESBL Production, and Antibiogram in a Tertiary Care Hospital in Bihar

**DOI:** 10.7759/cureus.115213

**Published:** 2026-08-26

**Authors:** Randhir Kumar, Nirmala Kumari, Kumar Saurabh, Kamlesh Rajpal, Shailesh Kumar, Namrata Kumari

**Affiliations:** 1 Microbiology, Indira Gandhi Institute of Medical Sciences, Patna, IND

**Keywords:** antibiogram, bihar, esbl, haemagglutination, haemolysin, uropathogenic e. coli, virulence factors

## Abstract

Background

Uropathogenic *Escherichia coli* (UPEC) is responsible for 50-90% of all urinary tract infections (UTIs) worldwide, with increasing antimicrobial resistance posing a significant public health threat. This study aimed to characterise UPEC isolates from a tertiary care hospital in Bihar by detecting virulence factors and determining their antibiogram.

Methods

A total of 246 UPEC isolates were collected from outpatient department (OPD) and inpatient department (IPD) patients at IGIMS, Patna, over 12 months. Identification was confirmed by Matrix-Assisted Laser Desorption/Ionisation Time-of-Flight Mass Spectrometry (MALDI-TOF MS) (Bruker Daltonics, Bremen, Germany). Virulence factors, including haemolysin, haemagglutination, and cell surface hydrophobicity, were assessed. Extended-spectrum beta-lactamase (ESBL) production was confirmed by a phenotypic combination of disk diffusion test.

Results

Of 246 isolates, females predominated (52.0%). Haemolysin was detected in 44 (17.9%) isolates, haemagglutination in 30 (12.2%), cell surface hydrophobicity in 13 (5.3%), and ESBL production in 15 (6.1%). Most isolates showed lactose fermenting (LF) colonies on CLED (80.1%). High resistance rates to ampicillin and co-trimoxazole were noted. Nitrofurantoin and gentamicin retained better activity.

Conclusion

UPEC isolates from IGIMS, Patna, showed considerable virulence factor prevalence and antimicrobial resistance. Nitrofurantoin and aminoglycosides remain useful therapeutic options. Routine characterisation of UPEC is essential for effective UTI management in this region.

## Introduction

Urinary tract infection (UTI) is one of the most common bacterial infections encountered in both community and hospital settings, affecting individuals across all age groups. *Escherichia coli* (*E. coli*) is the predominant uropathogen, accounting for approximately 50-90% of uncomplicated UTIs worldwide [1]. Globally, nearly 150 million cases of UTI occur annually, resulting in substantial healthcare expenditure and significant morbidity, particularly among women, elderly individuals, and patients with underlying comorbidities [2].

Uropathogenic *E. coli* (UPEC), a distinct pathotype of *E. coli*, possesses a variety of virulence factors that facilitate colonisation, persistence, and invasion of the urinary tract. These virulence determinants include adhesins such as fimbriae, haemolysin production, cell surface hydrophobicity, and the ability to evade host immune responses [3,4]. Haemolysin, particularly alpha-haemolysin, acts as a cytotoxin that damages host cells and contributes to tissue invasion and inflammation. Fimbrial adhesins can be detected phenotypically by haemagglutination assays using D-mannose, while cell surface hydrophobicity promotes bacterial adherence to uroepithelial cells and biofilm formation, thereby increasing bacterial persistence within the urinary tract [3,4].

The emergence and widespread dissemination of antimicrobial resistance among UPEC strains have become a major public health concern worldwide. Of particular importance is the production of extended-spectrum beta-lactamases (ESBLs), enzymes capable of hydrolyzing third-generation cephalosporins and other beta-lactam antibiotics, thereby limiting therapeutic options and contributing to treatment failure [5]. ESBL-producing UPEC isolates are frequently associated with multidrug resistance, prolonged hospital stays, increased healthcare costs, and adverse clinical outcomes.

According to the definitions proposed by the European Centre for Disease Prevention and Control (ECDC) and the United States Centers for Disease Control and Prevention (CDC), multidrug-resistant (MDR) organisms are defined as isolates demonstrating non-susceptibility to at least one agent in three or more antimicrobial classes [6]. Extensively drug-resistant (XDR) organisms remain susceptible to only one or two antimicrobial categories, whereas pandrug-resistant (PDR) organisms exhibit resistance to all available antimicrobial agents [7]. The increasing prevalence of MDR- and ESBL-producing UPEC strains has significantly complicated empirical treatment strategies for UTIs.

Bihar, one of the most densely populated states in eastern India, has limited published data regarding the virulence characteristics and antimicrobial susceptibility patterns of UPEC isolates. Tertiary care hospitals in the region are increasingly encountering infections caused by MDR UPEC strains, making local epidemiological surveillance essential for guiding empirical therapy and antimicrobial stewardship programs.

Therefore, the present study was undertaken at the Indira Gandhi Institute of Medical Sciences (IGIMS), Patna, Bihar, to isolate and characterise UPEC from patients with UTIs by evaluating major virulence factors, including haemolysin production, haemagglutination, and cell surface hydrophobicity; detecting ESBL production; and determining their antimicrobial susceptibility patterns. In addition, the study aimed to evaluate the association between virulence factors, ESBL production, demographic characteristics, and antimicrobial resistance patterns. The findings of this study may contribute to improved understanding of the local epidemiology of UPEC and support evidence-based empirical therapy, antimicrobial stewardship, and infection control practices in this region [8,9].

## Materials and methods

Study design and setting

This was an observational cross-sectional study conducted over 12 months (after IEC approval) in the Bacteriology section of the Department of Microbiology, IGIMS, Patna, Bihar, India. Clinical urine samples were collected from patients attending OPD and IPD of various departments based on relevant clinical signs and symptoms such as fever, burning micturition, dysuria, and frequency.

Ethical approval

The study was approved by the Institutional Ethics Committee (IEC) of IGIMS, Patna, Bihar, India (Approval No. 232/IEC/IGIMS/2024; Date: 19/10/2024). The study was conducted in accordance with the ethical principles of the Declaration of Helsinki. Written informed consent was obtained from all participants or their legally authorised representatives prior to sample collection.

Sample collection and processing

All morning midstream clean-catch urine samples showing a single morphotype colony of *E. coli* with a significant count (101-105 CFU/mL) from the microbiology laboratory were processed for the identification of UPEC. Samples with mixed growth or contaminated cultures were excluded. Patients with a recent history of antibiotic use prior to sample collection were also excluded.

Inclusion criteria

All uropathogenic *E. coli* isolates recovered from clinical urine samples with significant bacteriuria (≥105 CFU/mL) were included.

Sample size

A minimum sample size of 246 *E. coli* isolates was calculated at a 95% confidence level with 5% absolute precision. A total of 246 isolates were analysed.

Identification of *E. coli*

Identification was performed by Gram stain and standard biochemical tests (Indole, Mannitol motility, Citrate, TSI, and Urease). All manual identifications were confirmed by Proteomics - Matrix-Assisted Laser Desorption/Ionisation Time of Flight Mass Spectrometry (MALDI-TOF MS). Colonies were spotted on target plates and processed through the ionisation chamber for automated analysis.

Identification of virulence factors

Haemolysin

​​​​*E. coli* isolates were inoculated on 5% sheep blood agar plates and incubated at 37°C overnight. The test was considered haemolytic when a zone of lysis was observed around each colony on blood agar plates, indicating alpha-haemolysin production.

Haemagglutination

Fimbriated *E. coli* produces haemagglutination detectable by erythrocyte clumping. Blood was washed three times with normal saline, and a 3% erythrocyte suspension was prepared in phosphate-buffered saline (PBS; pH 7.4). *E. coli* grown on nutrient agar was inoculated into 5 mL of PBS pH 7.4. The procedure was performed on VDRL slides. Bacterial suspension (40 µL) was mixed with 40 µL of human blood and 40 µL of PBS with and without 3% D-mannose. Slides were rotated on a VDRL rotator for 4 minutes and the reaction recorded. Both mannose-sensitive (MSHA) and mannose-resistant (MRHA) haemagglutination were noted.

Cell Surface Hydrophobicity (Salt Aggregation Test)

*E. coli* grown on nutrient agar was inoculated into 1 mL of PBS (pH 6.8) to achieve a colony count of 5 × 109 colonies/ml. Different molar concentrations of ammonium sulphate (1M, 1.4M, and 2M) were tested. Bacteria aggregating with salt particles were considered hydrophobic. The test was performed on VDRL slides.

Phenotypic confirmation of ESBL production

ESBL production was confirmed by the Combination Disk Diffusion Test (CDDT). Based on VITEK 2 results, isolates with MIC ≥ 16 mg/mL for ceftazidime were subjected to CDDT. A ceftazidime 30-mg disk (Becton Dickinson, Franklin Lakes, New Jersey) and a combination ceftazidime + clavulanic acid 30+10 mg disk (HiMedia Laboratories Pvt. Ltd., Mumbai, Maharashtra) were placed 60 mm apart and incubated at 37°C for 24 hours. A difference in zone size of >5 mm between the ceftazidime + clavulanic acid disk and the ceftazidime disk was confirmatory for ESBL production, per CLSI guidelines. Controls used were *E. coli* ATCC 25922 (ESBL-negative) and *Klebsiella pneumoniae* ATCC 700603 (ESBL-positive).

Antimicrobial susceptibility testing (AST)

AST was performed using Kirby-Bauer Disc Diffusion and VITEK 2 (bioMérieux India Pvt Ltd) panels. A panel of 27 antibiotics was tested, including Cefoxitin (CX), Ceftazidime (CAZ), Cefotaxime (CTX), Cefuroxime (CFR), Cefixime (CFM), Ceftriaxone (CTR), Ciprofloxacin (CIP), Clindamycin (CD), Aztreonam (AT), Azithromycin (AT), Tetracycline (TE), Oxacillin (OX), Gentamicin (GEN), Erythromycin (E), Levofloxacin (LE), Clarithromycin (CLR), Minocycline (MI), Imipenem (IMP), Amoxicillin/clavulanic acid (AMC), Ampicillin/Sulbactam (A/S), Amikacin (AK), Trimethoprim (COT), Colistin (CL), Tigecycline (TG), Chloramphenicol (C), Moxifloxacin (MOX), and Nitrofurantoin (NIT), as per standard protocol.

Statistical analysis

Data were entered into Microsoft Excel (Redmond, Washington) and analysed using IBM SPSS Statistics for Windows, Version 21 (Released 2012; IBM Corp., Armonk, New York). Categorical variables were expressed as frequencies and percentages. Associations between virulence factors (haemolysin production, haemagglutination, and cell surface hydrophobicity), ESBL production, demographic variables, and antimicrobial resistance patterns were analysed using the Chi-square test or Fisher's exact test, as appropriate. A two-tailed p-value of <0.05 was considered statistically significant.

## Results

Demographic profile

A total of 246 uropathogenic *E. coli* isolates were analysed. All were confirmed by MALDI-TOF MS. Females constituted 128 (52.0%) of the cases, while males accounted for 118 (48.0%), indicating a female preponderance. Age ranged from 1 to 81 years, with a mean age of 44.1 years. The highest number of isolates was from the age group 46-60 years (65; 26.5%), followed by 61 years and above (60; 24.4%). Table 1 shows the age-sex distribution. The age- and sex-wise distribution of UPEC isolates is presented in Table 1.

**Table 1:** Age-sex distribution of uropathogenic Escherichia coli (UPEC) isolates (n = 246).

Age group (years)	Female n (%)	Male n (%)	Total n (%)
0-14	9 (47.4%)	10 (52.6%)	19 (7.7%)
15-30	31 (58.5%)	22 (41.5%)	53 (21.5%)
31-45	30 (61.2%)	19 (38.8%)	49 (19.9%)
46-60	35 (53.8%)	30 (46.2%)	65 (26.4%)
61 and above	23 (40.4%)	37 (61.7%)	60 (24.4%)
Total	128 (52.0%)	118 (48.0%)	246 (100%)

Departmental distribution

The majority of isolates were from OPD/Urology (68; 27.6%), followed by OPD/General Medicine (46; 18.7%), OPD/Obstetrics & Gynaecology (41; 16.7%), and IPD (40; 16.3%). Table 2 presents the departmental distribution.

**Table 2:** Departmental distribution of UPEC isolates (n = 246). UPEC: uropathogenic *Escherichia coli*.

Department	Number of isolates	Percentage (%)
OPD/Urology	68	27.6%
OPD/General Medicine	46	18.7%
OPD/Obstetrics and Gynaecology	41	16.7%
IPD (Indoor)	40	16.3%
OPD/Nephrology	16	6.5%
OPD/Paediatrics	11	4.5%
Others	24	9.7%
Total	246	100%

Colony morphology on CLED

On CLED agar, 197 (80.1%) isolates produced lactose-fermenting (LF) colonies, and 49 (19.91%) were non-lactose-fermenting (NLF). Table 3 shows the colony morphology distribution. The distribution of colony morphology patterns is shown in Table 3.

**Table 3:** Colony morphology of UPEC on CLED agar (n = 246). UPEC: uropathogenic *Escherichia coli*.

Colony morphology on CLED	Number	Percentage (%)
Lactose fermenting (LF)	197	80.1%
Non-lactose fermenting (NLF)	49	19.9%
Total	246	100%

Virulence factors

Among 246 UPEC isolates, haemolysin was the most prevalent virulence factor, detected in 44 (17.9%) isolates. Haemagglutination was positive in 30 (12.2%) isolates. Cell surface hydrophobicity (salt aggregation test) was positive in 13 (5.3%) isolates. ESBL production was phenotypically confirmed in 15 (6.1%) isolates. The prevalence of the evaluated virulence factors is presented in Table 4.

**Table 4:** Prevalence of virulence factors in UPEC isolates (n = 246). UPEC: uropathogenic *Escherichia coli*.

Virulence factor	Positive n (%)	Negative n (%)	Total
Haemolysin (alpha-haemolysin)	44 (17.9%)	202 (82.1%)	246
Haemagglutination	30 (12.2%)	216 (87.8%)	246
Cell Surface Hydrophobicity (Salt Aggregation)	13 (5.3%)	233 (94.7%)	246
ESBL Production (Phenotypic)	15 (6.1%)	231 (93.9%)	246

Association between virulence factors and ESBL production

The association between each virulence factor (haemolysin, haemagglutination, and cell surface hydrophobicity) and ESBL production was assessed using the Chi-square test, with Fisher’s exact test applied where expected cell counts were less than 5. Results are summarised in Table 5.

**Table 5:** Association between virulence factors and ESBL production (n = 246).

Virulence factor	ESBL positive n (%) (n = 15)	ESBL negative n (%) (n = 231)	Test statistic (χ2/Fisher’s)	p-value
Haemolysin positive (n = 44)	4 (9.1%)	40 (90.9%)	NA (Fisher's exact)	0.318
Haemagglutination positive (n = 30)	6 (20.0%)	24 (80.0%)	NA (Fisher's exact)	0.005
Cell Surface Hydrophobicity positive (n = 13)	4 (30.8%)	9 (69.2%)	NA (Fisher's exact)	0.005

Association between ESBL production and demographic variables

The association between ESBL production and demographic variables (sex and age group) was assessed using the Chi-square test or Fisher’s exact test, as appropriate. Results are shown in Table 6.

**Table 6:** Association between ESBL production and demographic variables (n = 246). ESBL: extended-spectrum beta-lactamase.

Variable	Category	ESBL positive n (%)	ESBL negative n (%)	p-value
Sex	Female (n = 128)	9 (7.0%)	119 (93.0%)	0.711
	Male (n = 118)	6 (5.1%)	112 (94.9%)	-
Age group (years)	0-14 (n = 19)	1 (5.3%)	18 (94.7%)	0.313
	15-30 (n = 53)	5 (9.4%)	48 (90.6%)	-
	31-45 (n = 49)	0 (0.0%)	49 (100.0%)	-
	46-60 (n = 65)	4 (6.2%)	61 (93.8%)	-
	61 and above (n = 60)	5 (8.3%)	55 (91.7%)	-

Antibiogram

**Figure 1:**
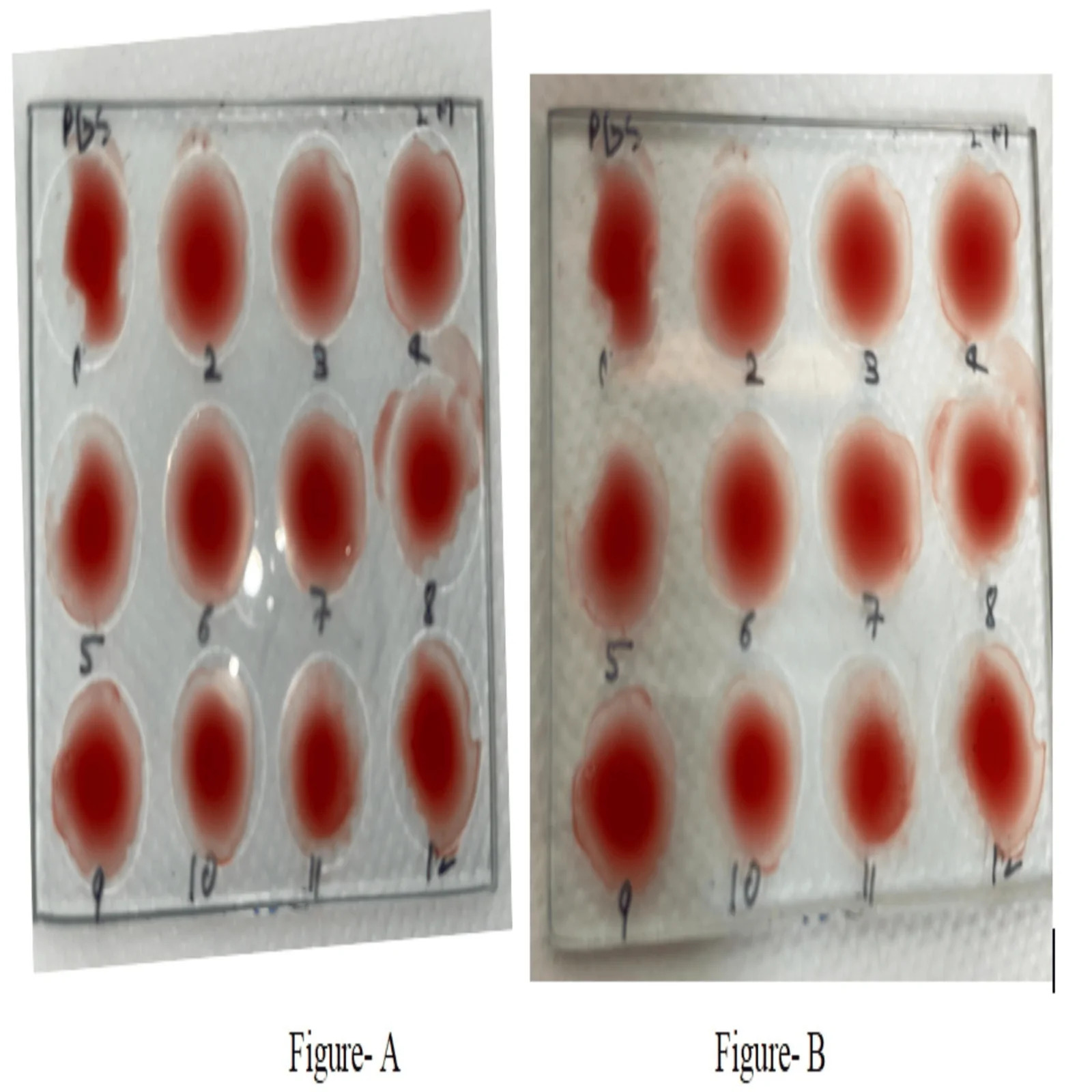
Haemagglutination test on VDRL slides. (A) Slide agglutination showing mannose-sensitive haemagglutination (MSHA) with clumping of erythrocytes in wells labelled 1-12. (B) Haemagglutination pattern with PBS and D-mannose controls; labelled wells 1-12 show gradations of agglutination. PBS: phosphate-buffered saline.

**Figure 2:**
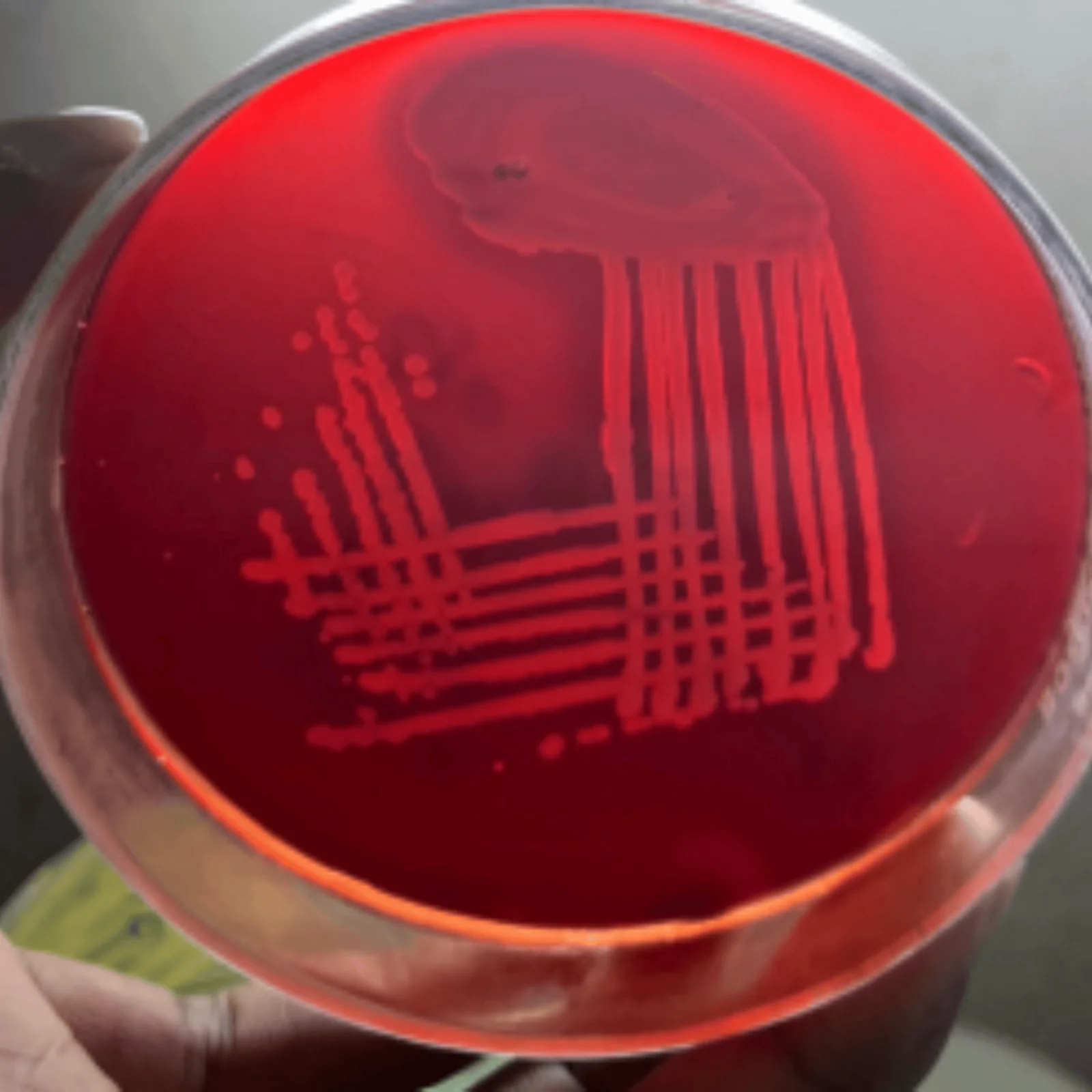
Phenotypic detection of haemolysin production in UPEC isolates on 5% sheep blood agar. Clear zones of haemolysis surrounding bacterial colonies indicate positive haemolysin production following overnight incubation at 37°C. UPEC: uropathogenic *Escherichia coli*.

**Figure 3:**
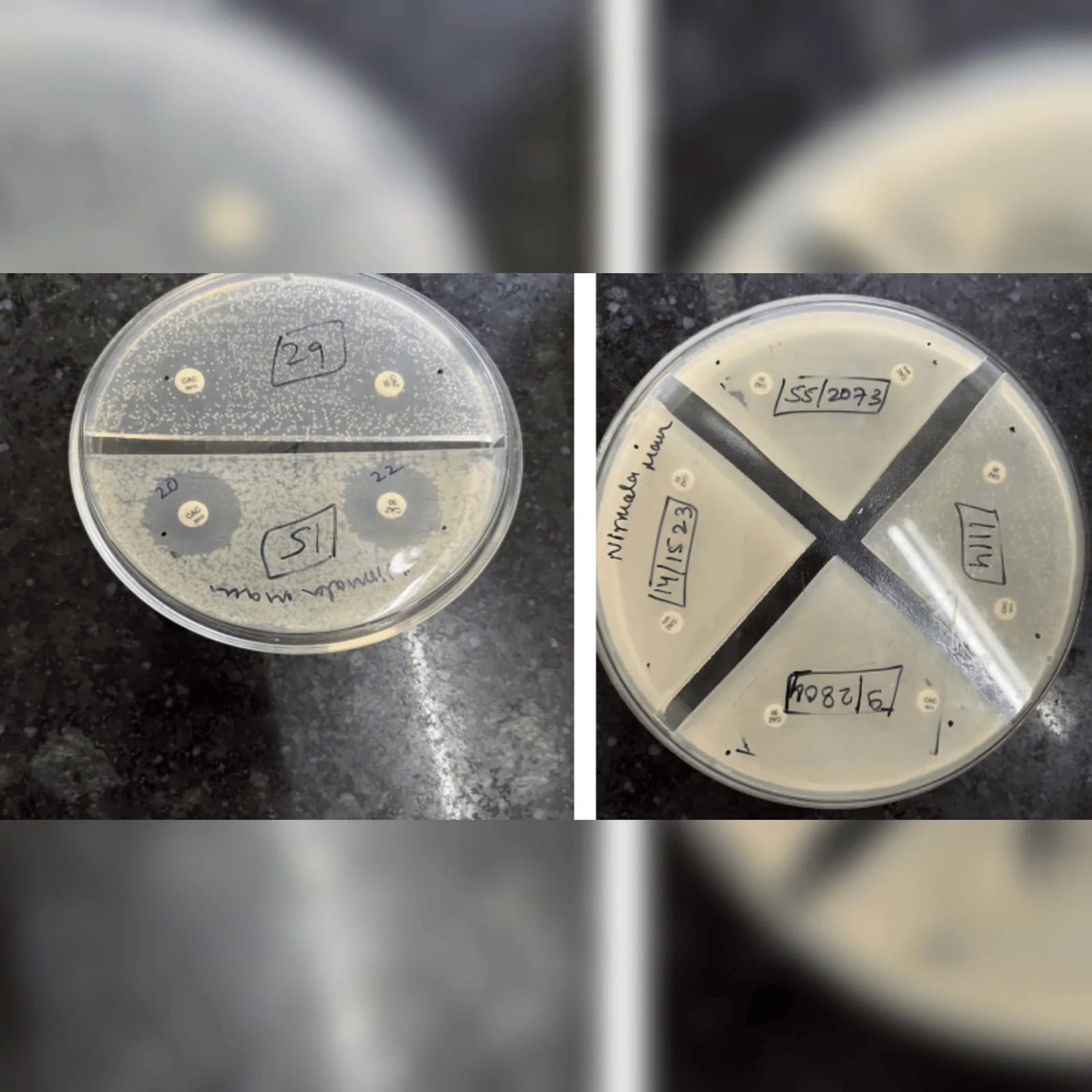
Combination Disk Diffusion Test (CDDT) for phenotypic confirmation of ESBL production. (A) A plate with two quadrants (isolates 29 and 15) demonstrating clear zone-of-inhibition enhancement around CAC disks in ESBL-positive isolates (lower quadrants labelled 20 mm and 27 mm). (B) A plate with four quadrants showing no clear zone-of-inhibition enhancement around CAC disc. ESBL: extended-spectrum beta-lactamase; CAC: ceftazidime-clavulanic acid.

Antimicrobial susceptibility testing revealed a high rate of resistance to ampicillin (AMP) and co-trimoxazole (COT) across the majority of isolates (Table 7). Resistance to fluoroquinolones (ciprofloxacin and levofloxacin) was also commonly observed. Nitrofurantoin (NIT) and gentamicin (GEN) retained comparatively better activity and were among the most frequently listed antibiotics in the susceptible category. Piperacillin-tazobactam (PIT) was susceptible in a significant proportion of isolates. Carbapenems (imipenem, meropenem, and ertapenem) remained active in most isolates, but resistance was recorded in some MDR cases. Colistin (CL) showed activity in the XDR subset. Multiple drug resistance patterns were noted, especially in isolates from IPD and ICU settings. Representative haemagglutination patterns observed during the study are shown in Figure 1, demonstrating both positive haemagglutination reactions and the corresponding PBS and D-mannose control wells. Haemolysin production by UPEC isolates was detected by the presence of clear zones of haemolysis surrounding colonies on 5% sheep blood agar. Representative haemolytic colonies are illustrated in Figure 2. Phenotypic confirmation of ESBL production was performed using the CDDT. ESBL-positive isolates demonstrated a ≥5 mm increase in the zone diameter around ceftazidime-clavulanic acid (CAC) disks compared with ceftazidime alone, whereas ESBL-negative isolates showed no appreciable enhancement in inhibition zones. Representative CDDT results illustrating both ESBL-positive and ESBL-negative patterns are shown in Figure 3.

**Table 7:** Complete antibiogram of UPEC isolates (n = 246). UPEC: uropathogenic *Escherichia coli*.

Antibiotic	Sensitive n (%)	Intermediate n (%)	Resistant n (%)
Ampicillin (AMP)	40 (16.3%)	8 (3.3%)	198 (80.5%)
Cefoxitin (CX)	134 (54.5%)	12 (4.9%)	100 (40.7%)
Ceftazidime (CAZ)	128 (52.0%)	12 (4.9%)	106 (43.1%)
Cefotaxime (CTX)	115 (46.7%)	13 (5.3%)	118 (48.0%)
Cefuroxime (CFR)	129 (52.4%)	7 (2.8%)	110 (44.7%)
Cefixime (CFM)	124 (50.4%)	17 (6.9%)	105 (42.7%)
Ceftriaxone (CTR)	126 (51.2%)	11 (4.5%)	109 (44.3%)
Ciprofloxacin (CIP)	90 (36.6%)	8 (3.3%)	148 (60.2%)
Clindamycin (CD)	53 (21.5%)	9 (3.7%)	184 (74.8%)
Aztreonam (AT)	120 (48.8%)	16 (6.5%)	110 (44.7%)
Azithromycin (AZM)	77 (31.3%)	15 (6.1%)	154 (62.6%)
Tetracycline (TE)	97 (39.4%)	11 (4.5%)	138 (56.1%)
Oxacillin (OX)	49 (19.9%)	17 (6.9%)	180 (73.2%)
Gentamicin (GEN)	164 (66.7%)	8 (3.3%)	74 (30.1%)
Erythromycin (E)	66 (26.8%)	13 (5.3%)	167 (67.9%)
Levofloxacin (LE)	78 (31.7%)	6 (2.4%)	162 (65.9%)
Clarithromycin (CLR)	68 (27.6%)	18 (7.3%)	160 (65.0%)
Minocycline (MI)	140 (56.9%)	13 (5.3%)	93 (37.8%)
Imipenem (IMP)	177 (72.0%)	11 (4.5%)	58 (23.6%)
Amoxicillin/Clavulanic acid (AMC)	114 (46.3%)	19 (7.7%)	113 (45.9%)
Ampicillin/Sulbactam (A/S)	108 (43.9%)	14 (5.7%)	124 (50.4%)
Amikacin (AK)	150 (61.0%)	13 (5.3%)	83 (33.7%)
Co-trimoxazole/Trimethoprim (COT)	49 (19.9%)	10 (4.1%)	187 (76.0%)
Colistin (CL)	198 (80.5%)	11 (4.5%)	37 (15.0%)
Tigecycline (TG)	189 (76.8%)	10 (4.1%)	47 (19.1%)
Chloramphenicol (C)	122 (49.6%)	16 (6.5%)	108 (43.9%)
Moxifloxacin (MOX)	90 (36.6%)	10 (4.1%)	146 (59.3%)
Nitrofurantoin (NIT)	163 (66.3%)	11 (4.5%)	72 (29.3%)
Piperacillin-Tazobactam (PIT)	158 (64.2%)	11 (4.5%)	77 (31.3%)

Association between ESBL production and antimicrobial resistance

Resistance rates to key antimicrobial agents were compared between ESBL-producing and non-ESBL-producing isolates using the Chi-square test or Fisher’s exact test, as appropriate. Results for representative agents across the major antibiotic classes tested are presented in Table 8.

**Table 8:** Association between ESBL production and resistance to representative antimicrobial agents. ESBL: extended-spectrum beta-lactamase.

Antibiotic	Resistant among ESBL positive n (%) (n = 15)	Resistant among ESBL negative n (%) (n = 231)	p-value
Ampicillin	14 (93.3%)	184 (79.7%)	0.315
Co-trimoxazole	13 (86.7%)	174 (75.3%)	0.532
Ciprofloxacin	12 (80.0%)	136 (58.9%)	0.178
Levofloxacin	11 (73.3%)	151 (65.4%)	0.727
Gentamicin	5 (33.3%)	69 (29.9%)	0.776
Amikacin	7 (46.7%)	76 (32.9%)	0.417
Piperacillin-Tazobactam	6 (40.0%)	71 (30.7%)	0.566
Imipenem	3 (20.0%)	55 (23.8%)	1.000
Colistin	1 (6.7%)	36 (15.6%)	0.707
Nitrofurantoin	4 (26.7%)	68 (29.4%)	1.000

Multidrug-resistant prevalence

Applying the CDC/ECDC criteria described in the Introduction (non-susceptibility to at least one agent in three or more antimicrobial categories), 96 of 246 isolates (39.0%) were classified as MDR. Nine isolates (3.7%) met criteria for XDR, and two isolate(s) (0.8%) were pan-drug-resistant (PDR). MDR was numerically more frequent among isolates from IPD/ICU settings compared with OPD, consistent with the antibiogram findings above (Table 9).

**Table 9:** Multidrug-resistant (MDR) prevalence by care setting (n = 246). MDR: multidrug-resistant.

Care setting	MDR positive n (%)	Non-MDR n (%)	p-value
OPD (n = 182)	58 (31.9%)	124 (68.1%)	<0.001
IPD (n = 40)	33 (82.5%)	7 (17.5%)	-
Total (n = 222)	91 (41.0%)	131 (59.0%)	

Association between MDR, ESBL production, and virulence factors

The association between MDR status and ESBL production, as well as between MDR status and each virulence factor, was assessed using the Chi-square test or Fisher’s exact test, as appropriate. Results are presented in Table 10.

**Table 10:** Association between MDR status, ESBL production, and virulence factors (n = 246). MDR: multidrug-resistant; ESBL: extended-spectrum beta-lactamase.

Variable	MDR positive n (%)	Non-MDR n (%)	p-value
ESBL positive (n = 15)	11 (73.3%)	4 (26.7%)	0.011
ESBL negative (n = 231)	85 (36.8%)	146 (63.2%)	-
Haemolysin positive (n = 44)	20 (45.5%)	24 (54.5%)	0.427
Haemolysin negative (n = 202)	76 (37.6%)	126 (62.4%)	-
Haemagglutination positive (n = 30)	16 (53.3%)	14 (46.7%)	0.130
Haemagglutination negative (n = 216)	80 (37.0%)	136 (63.0%)	-
Cell Surface Hydrophobicity Positive (n = 13)	8 (61.5%)	5 (38.5%)	0.156
Cell Surface Hydrophobicity Negative (n = 233)	88 (37.8%)	145 (62.2%)	-

The present study provides important regional epidemiological data on the phenotypic virulence characteristics, ESBL production, and antimicrobial susceptibility patterns of UPEC isolates from a tertiary care hospital in Bihar, where published data remain limited. Unlike many previous studies that have focused primarily on antimicrobial susceptibility or individual virulence determinants, the present study comprehensively evaluated multiple phenotypic virulence factors together with ESBL production and antibiogram patterns in a single cohort of UPEC isolates. Furthermore, the study provides valuable local evidence on the prevalence of virulence factors and antimicrobial resistance that can support empirical antibiotic selection, antimicrobial stewardship initiatives, and future surveillance programmes in this region.

## Discussion

This study characterised 246 UPEC isolates from the IGIMS, Patna, a tertiary care hospital in Bihar, by evaluating virulence factors and antimicrobial susceptibility patterns. Females constituted the majority of cases (52.0%), which is consistent with the well-established higher susceptibility of women to UTIs due to anatomical factors including a shorter urethra and its proximity to the perineum [1]. The highest number of isolates was observed in the 46-60 years age group, likely reflecting the increasing prevalence of comorbid conditions such as diabetes mellitus and prostatic enlargement in this age group, which predisposes individuals to recurrent and complicated UTIs.

Haemolysin production was detected in 17.9% of UPEC isolates in the present study, a finding comparable to those reported from other tertiary care centres in India [1,9,10]. Alpha-haemolysin is an important pore-forming cytotoxin that contributes to erythrocyte lysis, induction of inflammatory responses, tissue damage, and invasion of uroepithelial cells, thereby facilitating ascending UTIs and pyelonephritis [2,3]. The prevalence of haemolysin production reported in the literature generally ranges from 18% to 32%, supporting its role as one of the most consistently expressed virulence determinants among UPEC isolates [9,10].

Haemagglutination was observed in 12.2% of isolates. Type 1 fimbriae, which mediate mannose-sensitive haemagglutination, and P fimbriae, which mediate mannose-resistant haemagglutination, are important adhesins that facilitate attachment of UPEC to uroepithelial cells [2]. In the present study, both mannose-sensitive and mannose-resistant haemagglutination patterns were evaluated to identify fimbrial expression. The lower prevalence observed compared with some previous studies may reflect differences in bacterial strain characteristics, geographical variation, and laboratory culture conditions affecting fimbrial expression [9,10]. Earlier studies have reported haemagglutination positivity ranging from 40% to over 50% among UPEC isolates [9,10].

Cell surface hydrophobicity assessed by the salt aggregation test was positive in 5.3% of isolates. Hydrophobicity contributes to bacterial adherence, colonisation, and persistence on uroepithelial surfaces and is considered an important virulence characteristic of UPEC [8]. The prevalence observed in the present study was lower than that reported in several previous investigations, where positivity rates of approximately 20-25% were documented [10]. Such variations may be attributable to differences in isolate origin, patient populations, and methodological approaches used for detection.

Phenotypic confirmation of ESBL production was obtained in 6.1% of isolates using the combination disk diffusion test. The relatively lower prevalence of ESBL production compared with some reports from India may be explained by the predominance of outpatient isolates in the study population. Nevertheless, ESBL-producing isolates demonstrated resistance to multiple antimicrobial classes, with susceptibility frequently retained only to carbapenems and colistin in selected cases, emphasising the clinical challenges posed by these organisms [5,6].

Antimicrobial susceptibility testing revealed high resistance rates to ampicillin and co-trimoxazole, findings that are consistent with reports from several regions of India and other developing countries [1,4]. Resistance to fluoroquinolones was also considerable, likely reflecting their prolonged and widespread empirical use in the treatment of UTIs. Nitrofurantoin retained comparatively high activity against UPEC isolates and continues to be recommended as an effective first-line agent for uncomplicated lower UTIs caused by *E. coli*, particularly when renal function is adequate. Piperacillin-tazobactam also demonstrated satisfactory activity against many isolates. Among the aminoglycosides, gentamicin exhibited better susceptibility rates than amikacin in the present study. Although carbapenem resistance was detected in only a minority of isolates, its presence remains concerning because these agents are often considered drugs of last resort. High susceptibility rates to nitrofurantoin among ESBL-producing and MDR isolates have also been reported previously, supporting its continued role as a reliable empirical therapeutic option for lower UTIs in the Indian setting [7,11].

The relationship between virulence determinants and antimicrobial resistance remains an area of ongoing investigation. Previous studies have suggested that antimicrobial resistance and virulence traits may coexist within the same bacterial populations owing to co-selection mechanisms and shared mobile genetic elements [5,9]. However, the present study did not perform statistical analysis to establish a significant association between virulence factor expression and antimicrobial resistance patterns. Further large-scale studies incorporating molecular methods are required to better understand the interaction between virulence and resistance determinants in UPEC isolates. The differences observed between the present study and previous reports may be attributed to variations in geographic location, patient demographics, healthcare settings, antimicrobial prescribing practices, laboratory methodologies, and the predominance of outpatient isolates in our study population. These regional variations emphasise the importance of continuous local surveillance to guide empirical therapy and infection control practices.

The present study has several limitations. It was conducted at a single tertiary care centre, which may limit the generalisability of the findings to other healthcare settings and geographic regions. In addition, molecular characterisation of virulence genes and resistance determinants, including genes such as hlyA, papC, fimH, and iutA, was not performed. Future studies incorporating polymerase chain reaction (PCR)-based methods and whole genome sequencing may provide a more comprehensive understanding of virulence-resistance interactions and the molecular epidemiology of UPEC isolates in Bihar and surrounding regions. In addition, the study relied primarily on phenotypic methods and did not evaluate the genetic basis of antimicrobial resistance or virulence determinants, which may have provided deeper insights into the epidemiology of UPEC isolates.

## Conclusions

This study demonstrates that UPEC isolates from a tertiary care hospital in Bihar exhibit varying prevalence of phenotypic virulence factors, ESBL production, and antimicrobial resistance. Nitrofurantoin and piperacillin-tazobactam retained favourable activity against many isolates and may remain useful therapeutic options based on local susceptibility patterns. As this was a single-centre descriptive cross-sectional study, the findings primarily reflect the local epidemiology of UPEC in our setting and should be interpreted accordingly. Further multicentre studies incorporating molecular characterisation of virulence and resistance determinants, along with clinical outcome correlations, are warranted to validate and extend these findings.
